# End-of-life cohorts from the Dartmouth Institute: risk adjustment across health care markets, the relative efficiency of chronic illness utilization, and patient experiences near the end of life

**DOI:** 10.1007/s43999-024-00039-9

**Published:** 2024-03-29

**Authors:** Kristen K. Bronner, David C. Goodman

**Affiliations:** The Dartmouth Institute for Health Policy & Clinical Practice, Geisel School of Medicine at Dartmouth, Williamson Translational Research Building, Level 5, 1 Medical Center Drive, Lebanon, NH 03756 United States

**Keywords:** End-of-life care, Risk adjustment, Unwarranted regional variation, Small area analysis, Dartmouth Atlas

## Abstract

Since their inception, small area studies intended to measure health system performance have been challenged by concerns that regional variation in health care may be primarily explained by differences in patient health risk. Controlling for regional population differences depends on appropriate risk adjustment, but the adequacy of the methods used in early analyses was contested. A novel response to these concerns was the development of end-of-life cohorts by Dartmouth Atlas investigators. These were used initially to control for differences in population health status in studies investigating relative efficiency across regions. Later, they became useful for studying hospital-level variation in chronic illness care, and for measuring utilization and patient experiences at the very end of life. Altogether, end-of-life cohorts have been invaluable for clarifying the contribution of health system and provider factors to health care variation and outcomes.

## Introduction

By the early 1990s, geographic variation in health care delivery was a well-established field of academic inquiry and policy discussion [1, 2]. In the preceding two decades, researchers had observed large differences in utilization and spending for health services of all types—medical and surgical, inpatient and outpatient, primary and specialty care—in the United States and other countries, and across large and small areal units, including states, counties, cities, and market-based health service areas [3–22]. The published literature had established unequivocally the occurrence of health care variation, although the magnitude and causes remained a matter of debate [23–26]. Was the variation explained by regional differences in the prevalence of illness and patient preferences, or by varying clinician practice styles? Did people living in areas where less medical care was delivered receive the care they needed and wanted, or was there evidence of under-service? Did people living in places with higher health care capacity, utilization, and spending live longer and have better quality of life? Or was there evidence of overservice?

Studies designed to explore these questions were often criticized on the grounds that the methods did not adequately adjust for differences across regional populations. Researchers employed several approaches to control for population differences, including analyzing relatively homogeneous [5, 27, 28] or demographically similar populations [18, 29], restricting studies to patients with the same illnesses [22, 30–32], and adjusting for case mix [10, 17, 33, 34]. As the scope of the research expanded from regional to national populations, the need for better risk adjustment grew. Some health systems and researchers argued that the risk adjustment methods used to date were inadequate, offering evidence that variation in medical care could be explained largely by patient need [20, 35–39].

The use of end-of-life cohorts by Dartmouth Atlas authors originated from the necessity of better risk adjustment; a secondary application was to measure care at the very end of life to understand the use of curative and palliative interventions. This paper will examine the development and use of this approach, first to address the risk-adjustment problem; then to investigate the consequences of variation in elderly patients with chronic illness; and finally, to identify care for patients for whom higher medical service intensity is generally viewed as futile or even harmful. We will discuss Dartmouth papers primarily, but we will also include studies that challenged the Dartmouth approach or extended its applications and findings.

## Early Dartmouth end-of-life studies

Early variation studies from John Wennberg and his co-investigators were not primarily concerned with the specific issues of curative or palliative care in the elderly with advanced illness. However, several analyses used population mortality as a key measure to address the general question of whether residing in an area with higher-intensity health care was associated with better outcomes [5, 28]. Wennberg’s first small area analysis, published in *Science *in 1973, found no significant correlation between higher spending for hospital and physician services and age-adjusted mortality across 13 areas in the state of Vermont [5]. Subsequent research focused on Medicare beneficiaries living in the cities of Boston and New Haven and found that age, sex, and race-adjusted population mortality was not lower in Boston than in New Haven, despite the fact that beneficiaries in Boston had a 47 percent higher hospital discharge rate, 15 percent longer hospital stays, and 79 percent higher reimbursements per capita [19]. An earlier analysis of the two cities had attributed most of the higher utilization in Boston to the higher number of hospital beds per capita (4.5 per 1,000 residents, compared to 2.9 per 1,000 in New Haven) [18], foreshadowing a recurring theme of high capacity as a key driver of overuse.

**Fig. 1:**
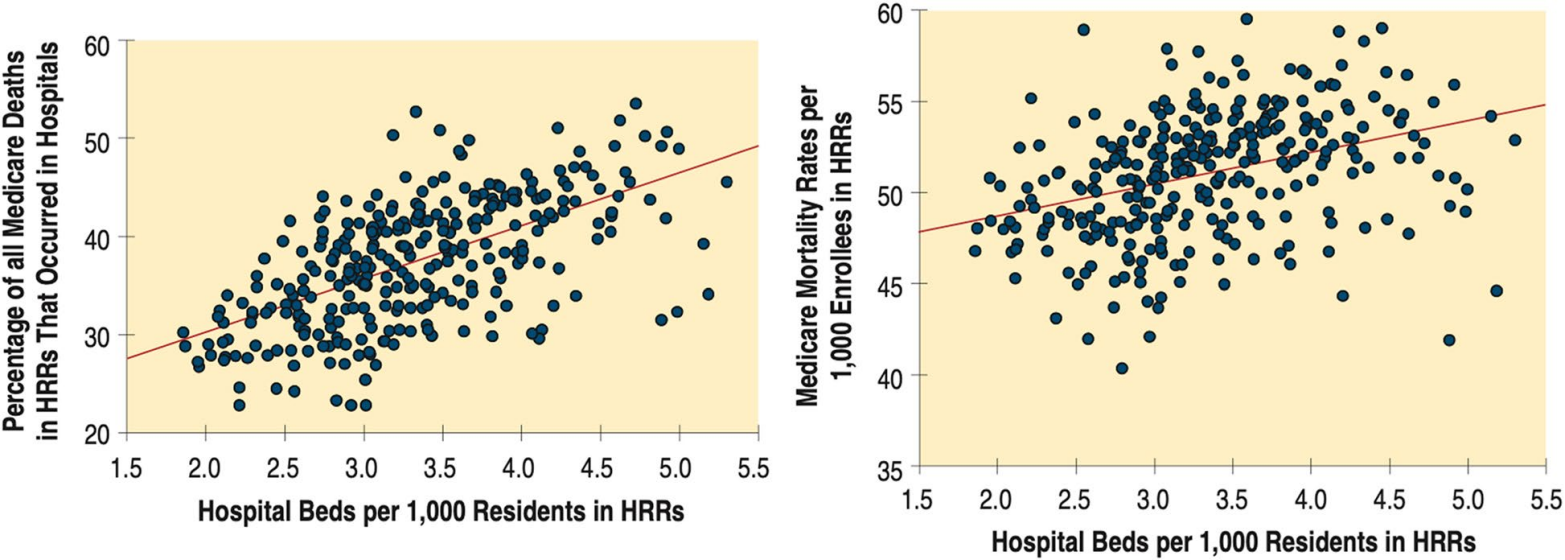
The Associations Between Allocated Hospital Beds and Measures of Hospital Death and Mortality [43]. Left Panel “Greater hospital capacity increases the likelihood that Medicare deaths will occur in the hospital (*R*^2^=.36). In the 33 regions with fewer than 2.5 beds per thousand residents, an average of 30.5% of all Medicare deaths occurred in a hospital. In the 21 regions with more than 4.5 beds per thousand residents, the rate was 1.52 times higher; an average of 46.6% of deaths occurred during a hospitalization.” (From “The Dartmouth Atlas of Health Care (1996).” Figure 3.6, page 54.). Right panel “Mortality rates, after adjustment for age, sex, and race, tend to be higher in regions with greater numbers of allocated beds (*R*^2^=.13). In the 33 regions with fewer than 2.5 beds per thousand residents, the population-weighted and age, sex and race adjusted mortality rate was 49.4; in the 21 regions with more than 4.5 beds, the rate was 51.2.” (From “The Dartmouth Atlas of Health Care (1996).” Figure 3.8, page 56.)

The first *Dartmouth Atlas of Health Care*, published in 1996, expanded the small area variation studies of the 1970s and 1980s to a national scale [40]. Building upon the Boston-New Haven and other studies [41, 42], the report used population-based mortality rates as an indicator to demonstrate the association—or lack thereof—between either utilization or capacity and population health outcomes. Figure 1 shows the relationship (*R*^2^=0.36) between the number of hospital beds per 1,000 in a hospital referral region and, among other utilization measures, the likelihood that Medicare patients who died would do so in a hospital (Fig. 1, left). As with other measures, the capacity of the local health care system was associated with the propensity to hospitalize patients, including those who were at the end of their lives. The same report also showed that not only did a higher number of beds correlate with a higher rate of in-hospital death; it also correlated with slightly *higher* population-based mortality (*R*^2^=0.13), which is consistent with the earlier finding that having more beds in a region does not necessarily result in lower mortality rates (Fig. 1, right) [43]. The association is also consistent with the view that regions with higher health burdens use more hospital beds to care for sicker patients. This ambiguity motivated future efforts by Atlas investigators to develop measures of care intensity that were not confounded by population need.

**Fig. 2:**
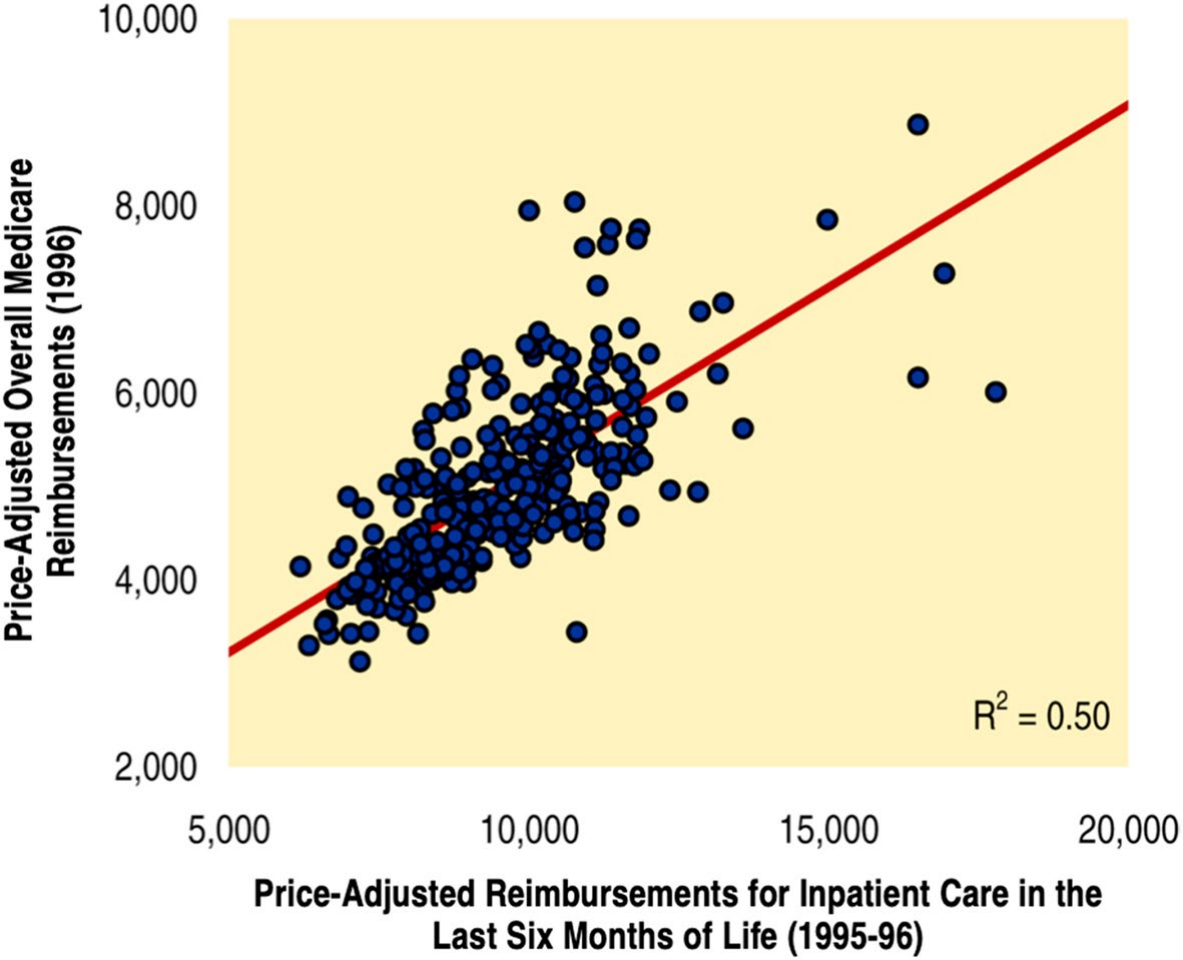
The Association Between Inpatient Medicare Spending in the Last Six Months of Life and Overall Per Capita Spending in the General Medicare Population [45]. “The intensity of care in the last six months of life, measured by Medicare Part A spending, is closely correlated with overall Medicare per capita spending (Part A and B) for the entire Medicare population.” (From “The Quality of Medical Care in the United States: A Report on the Medicare Program. The Dartmouth Atlas of Health Care 1999.” Figure 6.13, page 197.)

The next Atlas editions, released in 1998 and 1999, extended the analysis to include other regional measures of care intensity during the last six months of life, including the propensity to hospitalize and admit patients to intensive care units; the number of days patients spent in hospitals and ICUs; reimbursements for inpatient care and ICU charges per patient; the number of physician visits per patient; and the number of different physicians involved in patients’ care [44, 45]. The 1999 Atlas also documented the strong association (*R*^2^=0.50) between spending for inpatient care during the last six months of life and average per capita Medicare reimbursements for all enrollees (Fig. 2), reporting that “how people are treated in the last six months of their lives is a good indicator of the overall intensity of medical intervention in the population” [45].

These early Atlas analyses were based on all Medicare patients who died, regardless of disease status or illness progression. Unlike earlier small area studies, which compared the care of patients in relatively localized regions with similar demographics, Medicare patients across the United States were not comparable, even though their outcomes were the same—they had all died. The mixture of chronic illness and age of death in the patient population are two examples of important differences across regions and hospitals; variation in the mix could affect both utilization and spending, as discussed below. Further control of patient factors was necessary for unbiased inference.

A subsequent study by Fisher et al published in the journal *Health Services Research *further explored the relationship between local hospital capacity, mortality, and the likelihood of dying in the hospital, this time controlling for disease burden in addition to sociodemographic characteristics [46]. The methods used to adjust for differences in population health included restricting to patient cohorts for whom hospital admission could be considered a legitimate proxy for disease incidence unaffected by area hospital bed supply—those hospitalized for hip fracture, heart attack, stroke, or surgical treatment of colon or lung cancer [18, 22, 33]—as well as differences in hospital day rates during the last six months of life among patients who died, under the assumption that these patients would be similarly ill in terms of their risk of death. Subsequent to the index admission, the researchers found no mortality benefit from increased area bed availability or propensity to hospitalize, and a greater likelihood that, among patients who died, death would occur in an inpatient setting. This study represented the first peer-reviewed analysis from Dartmouth investigators to use a decedent cohort to adjust for health status. The estimates of illness risk, however, were still ecologic measures of regional levels of hospitalization, education, income, poverty status, and disability. Although studies showed that these were valid proxies for underlying disease burden [47, 48], the chance for residual confounding remained.

These early studies did not find benefits to Medicare patients of greater resource capacity, higher spending, or more care during the last months of life. However, the studies did reveal positive correlations between capacity and the tendency to use the hospital for end-of-life care; between capacity and mortality; and between Medicare reimbursements for patients during the last six months of life and for beneficiaries overall. The use of end-of-life patients as a novel cohort with homogeneous outcomes confirmed these findings. The intensity of end-of-life care thus came to be understood as a measure that reflected utilization and spending that was unrelated to patient demand (i.e., need or preferences). Because all the patients in this group had an equal risk of mortality, the need for risk adjustment was reduced, though not eliminated entirely. Patients at the end of life are heterogenous across regions and hospitals in their age, sex, and mixture of chronic illness, requiring different clinical care. This challenge in risk adjustment would be addressed through a combination of study design and statistical modelling.

## The evolution of Dartmouth end-of-life studies

**Fig. 3:**
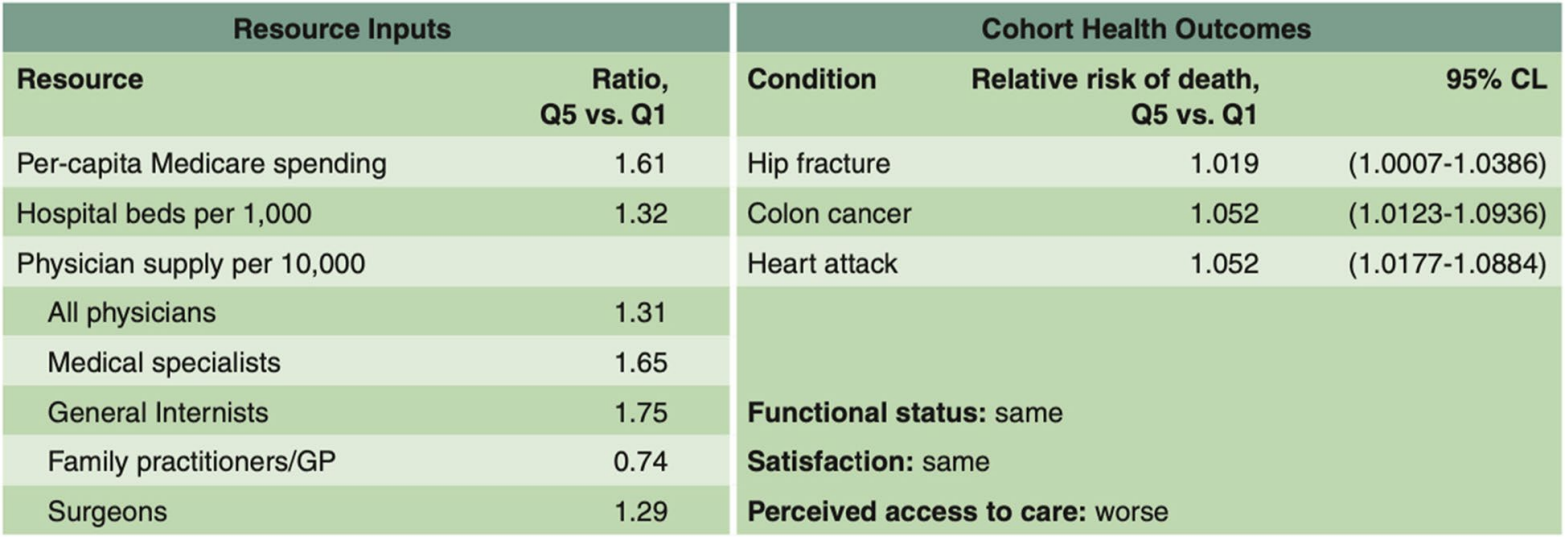
Per Capita Resource Inputs and Health Outcomes: The Ratio of High to Low Quintile of Spending [51]. “The table compares the level of resource inputs and mortality among cohorts living in hospital referral regions in the highest and lowest quintiles of Medicare end-of-life spending. The high input rate regions had 32% more hospital beds per capita, 31% more physicians, 65% more medical specialists, 75% more general internists, 29% more surgeons—and, of course, more Medicare spending (61% higher, on a price-adjusted basis). The low input rate regions had 26% more family practice physicians. Although the hip fracture, heart attack, and colon cancer cohorts were comparable in baseline morbidity over the five-year period of follow-up after the index event from which the diagnosis was made, those living in the high-rate regions had higher mortality rates: 1.9% higher for hip fracture patients, 5.2% higher for colon cancer patients, and 5.2% higher for heart attack patients.” (From “The Care of Patients with Severe Chronic Illness: A Report on the Medicare Program by the Dartmouth Atlas Project. The Dartmouth Atlas of Health Care 2006.” Table 1.1, page 4.) Adapted from Fisher et al, Annals of Internal Medicine, 2003 [49, 50]

In two papers published in the *Annals of Internal Medicine *in 2003 [49, 50], Fisher et al developed methods to use Medicare spending during the last six months of life as an exposure measure to examine whether patients living regions with higher intensity care received higher quality care or had better outcomes. First, each hospital referral region in the U.S. was assigned to a quintile of care intensity based on the End-of-Life Expenditure Index (EOL-EI), comprising Medicare spending on hospital and physician services per enrollee during their last six months of life. While exogenous to outcomes, it should be noted that the regional EOL-EI correlated highly with Medicare spending in the last two years of life. Second, cohorts of similarly ill patients—those hospitalized for hip fracture, colorectal cancer, or acute myocardial infarction—were assigned to their region of residence. Cohort characteristics were similar across the quintiles of EOL-EI. Medical care was then observed following their initial hospitalization. While patients living in regions with a high EOL-EI received 60 percent more care—largely explained by higher levels of inpatient-based and specialist-oriented care—they did not receive more recommended preventive services or appear to have greater access to care, [49] nor did they live longer, achieve better functional status, or express higher satisfaction with their care (Fig. 3) [50].

A subsequent study in *Health Affairs* applied the same cohorts and methodology to patients who were initially hospitalized and received most of their follow-up care at academic medical centers, finding similar results [52]; as was the case for hospital referral regions, high-intensity care at major teaching hospitals did not result in higher quality care or better survival. The authors concluded:

“Patients in the higher-intensity hospitals simply spend more time in the hospital and intensive care unit (ICU); have more frequent physician visits (especially in the inpatient setting); have more specialists involved in their care; and receive more imaging services, diagnostic testing, and minor (but not major) procedures. The similar results achieved with markedly different levels of resource inputs imply large differences in the longitudinal efficiency of chronic disease care across these hospitals.” [52]

In the same issue of *Health Affairs*, Wennberg et al demonstrated a method for measuring hospital-specific performance in managing chronically ill patients [53]. Building on research published earlier in the *British Medical Journal* [54], the researchers developed cohorts of patients with serious chronic illnesses associated with a high risk of in-hospital death [55]. Medicare patients who died with a diagnosis one of eleven chronic diseases were assigned to the hospital where they receive most of their care during the last two years of life. Utilization among the cohorts was further adjusted for the chronic illness case-mix; those who died without any evidence of chronic illness were excluded from the analysis. Both papers focused on seventy-seven hospitals that appeared on the 2001 *U.S.News and World Report *list of “America's best hospitals” for geriatric care, as well as care for cancer, heart, and lung disease [56].

The *British Medical Journal *paper documented twofold to sixfold variations in the use of medical care and resources to manage patients with chronic illness in the last six months of life among hospitals with reputations for high quality [54]. The *Health Affairs* paper demonstrated that the hospitals’ care patterns were consistent both for different types of patients and for earlier periods in patients’ lives. For example, utilization rates during the last six months of life were highly correlated among hospital-specific cohorts stratified by patients with cancer, congestive heart failure, and chronic obstructive pulmonary disease (Table 1); and Medicare spending per decedent during the last six months of life was closely related to per decedent spending during earlier phases of illness (Table 2) [53]. Another paper, focusing specifically on California regions and hospitals, extended the analysis to include a number of quality measures, derived from both claims data and technical quality measures posted on the Center for Medicare and Medicaid Services’ (CMS) Hospital Compare web site and summarized into quality scores by Jha et al, [57] as well as survey-based assessments of patient satisfaction with their hospital experiences [58]. Two utilization measures were portrayed as measures of care quality: the percent of patients admitted to intensive care units during the hospitalization in which they died, indicating aggressive terminal care; and the percent of patients seeing ten or more different physicians during the last six months of life, indicating the potential for poor communication and fragmented care [59]. The key findings included:

- The volume of services (hospital days per patient) was a more important factor than the price per service (price per day) in determining overall Medicare reimbursements per decedent (*R*^2^=0.65 for volume vs. 0.39 for price);
- The hospital most often used was a more important determinant of hospital day rates per patient than were age, race, gender, socioeconomic status, specific chronic illness, or severity of illness;
- Neither quality nor patient satisfaction was associated with higher utilization or spending [60].

**Table 1:** Correlations (R^2^) Between Utilization Rates during the Last Six Months of Life Among Chronically Ill Patient Cohorts Loyal to Seventy-Seven Selected Hospitals, 1999-2000 [53]

	**Hospital Days per Decedent**	**ICU Days per Decedent**	**Physician Visits per Decedent**
**Cohorts**			
Cancer & COPD	0.67	0.44	0.66
Cancer & CHF	0.64	0.46	0.69
CHF & COPD	0.85	0.85	0.70

The table illustrates the relationship (R^2^) between hospital-cohort specific utilization rates for three chronic illnesses during the last six months of life

*ICU* Intensive care unit, *COPD* Chronic obstructive pulmonary disease, *CHF* Congestive heart failure

**Table 2:** Correlations (R^2^) Between Medicare Reimbursements per Decedent during the Last Six Months of Life and Prior Intervals Before Death Among Chronically Ill Patient Cohorts Loyal to Seventy-Seven Selected Hospitals, 1999-2000 [53]

	**Medicare Reimbursements per Decedent**
**Months Before Death**	
0-6 & 7-12	0.85
0-6 & 13-18	0.79
0-6 & 19-24	0.70

The correlations show that illness-corrected estimates for spending during the last six months of life are highly correlated with patterns of practice in previous periods in the management of chronic illness

These studies advanced Dartmouth end-of-life research in several ways. First, they provided further evidence that end-of-life care intensity could be used as an independent proxy for the intensity of care overall at the regional and hospital level, not just care for dying patients. Second, they evolved the methods of risk adjustment by restricting the cohorts to patients who were similarly ill, minimizing the chances that unmeasured differences in illness levels explained the observed variations while not relying on potentially biased diagnostic coding practices. Third, they expanded the analyses from regions to specific hospitals—attributing patients to the hospitals where they actually received their care, not just to the regions in which they lived—allowing care intensity to be measured at the hospital level and focusing accountability for delivering that care on specific institutions. Finally, they introduced the strategy of evaluating the relative efficiency of medical centers and defining “best practice” benchmarks; hospitals that achieve equal or better outcomes while using fewer resources and spending less.

The 2006 edition of the *Dartmouth Atlas of Health Care* continued to explore these concepts, refining the methods of risk adjustment and broadening the scope to include data from not just selected regions or academic medical centers, but thousands of hospitals across the U.S. During the same period, additional peer-reviewed articles focused on specific topics in depth. A paper by Goodman et al published in *Health Affairs*and expanded upon in the 2006 Atlas examined physician workforce capacity—including the number of physicians per capita in a region, their work effort, and the relative proportions of primary care physicians and medical specialists—in end-of-life chronic illness cohorts, finding marked differences in hospital-level labor input (full-time equivalents per 1,000 beneficiaries) despite constant patient outcomes [51, 61]. Other studies continued to search for evidence that high-intensity end-of-life care was differentially preferred by patients in differing demographic or socioeconomic groups [62, 63], or that it was associated with higher quality [64].

A new exposure measure was introduced in the 2008 Atlas: the hospital care intensity (HCI) index, an age, sex, race, and illness-standardized score based on utilization—the number of days patients spent in the hospital and the number of physician visits they experienced as inpatients during the last two years of life [65]—rather than Medicare reimbursements, which require adjustments for regional costs of living, payments to hospitals with residency training programs, and payments to hospitals that serve a high percentage of low-income patients [66]. The HCI was used by Wennberg et al in a 2009 study in *Health Affairs *to compare the intensity of hospital care with measures of both technical quality and patient satisfaction, derived from the Hospital Consumer Assessment of Healthcare Providers and Systems (HCAHPS) survey and published on the CMS website [67]. The results showed that patients living in regions with high-intensity care tended to rate the hospitals they used poorly, both overall and on specific measures such as whether their pain was controlled, whether the staff communicated well, and whether their rooms were clean or quiet. Lower overall ratings were also correlated with lower technical quality scores for the care of heart attack, pneumonia, and congestive heart failure patients [68]. Researchers outside of Dartmouth have also used the HCI as an exposure measure for regional and hospital care intensity [69–74].

## Beyond risk adjustment: the experience of dying

Care at the end of life is not only important as a risk-adjusted proxy measure of care intensity; it is also of profound importance to the patient, family, and society. Unless death is the result of an unexpected catastrophic event, it is usually preceded by increased health care utilization intended to prolong life or improve its quality [75]. While assessing patient preferences is rarely straightforward, research has shown that patients do not generally prefer aggressive care in the face of serious chronic illness [62, 76–78], not only due to the challenges of disease progression and of medical treatments—such as pain, adverse events, and drug toxicity—but also the logistical burdens of increasingly intensive treatment such as frequent doctor visits and hospitalizations [79–81], along with higher copayments [82]. Though some patients may wish that everything possible be done to prolong their lives, most patients with a poor prognosis do not express a desire for life-sustaining treatment in the terminal phase of illness [83–85], nor is there evidence of a widespread preference to die in the hospital [86, 87].

Dartmouth investigators’ development of end-of-life cohorts for risk adjustment also generated descriptions of the variation in care near the very end of life that attracted widespread interest. The 2008 Atlas showed that, even among 93 prestigious hospitals included on the Council of Teaching Hospital’s list of integrated academic medical centers [88], the care delivered to patients with serious medical conditions varied remarkably. For example, among patients dying during the period from 2001 to 2005, the number of days spent in intensive care during the last six months of life varied more than eightfold, from 1.5 days per patient to 12.5, while the number of visits to medical specialists varied by a factor of nine, from 6.2 visits per patient to 55.8. There were also more than threefold variations in primary care visits per patient and in the percent of deaths that included an admission to intensive care [65]. In theory, these variations could be explained by patient preferences; however, in other studies using survey data, patient preferences have not been shown to be strongly associated with regional utilization and spending patterns [62, 89].

Later papers and reports concentrated on cohorts of cancer patients [90] and studied variations in the use of advanced life support interventions—such as endotracheal intubation, feeding tubes, and cardiopulmonary resuscitation—as well as in the use of palliative and hospice care for cancer patients with poor prognoses [91]. Little to no association was found between best practices in end-of-life cancer care and hospital characteristics such as membership in the National Comprehensive Cancer Network or designation as a comprehensive cancer center by the National Cancer Institute; in fact, uncomfortable treatments that are unlikely to prolong or enhance quality of life were more common in National Cancer Institute centers than in community hospitals [92]. A randomized clinical trial by Temel et al demonstrated that lung cancer patients receiving early palliative care integrated with standard oncologic care had better quality of life and longer survival—despite a lower likelihood of receiving aggressive end-of-life care—than those receiving standard oncologic care alone [93]. These papers extended the analysis to care that was not only potentially unnecessary, but arguably harmful.

## Criticisms of Dartmouth methods

As the Dartmouth Atlas became increasingly recognized as an important source of information regarding potentially unnecessary care and opportunities for saving money in the Medicare program, the methods and conclusions came under further scrutiny. Of particular concern was the use of end-of-life cohorts rather than forward-looking cohorts. Some critics argued that even looking backward from death could not ensure that the patients were similarly ill [94, 95], while others asserted that the research ignored those who may have survived because they received more care [96, 97], despite the lack of an association between greater care intensity and average population mortality.

To address these criticisms, Dartmouth researchers developed hospital-specific, risk-adjusted prospective cohorts of acute myocardial infarction patients and followed them forward for one year from their inception event (i.e., their heart attack), measuring both expenditures and mortality and correlating these indicators with the “look-back” reimbursement rates for patients in their last two years of life [98]. Despite the differences in method, the “look-forward” and “look-back” expenditure rates were highly correlated for 144 of the largest hospitals in the United States (*r*=0.85) and for all 2,360 hospitals in the sample (*r*=0.73), reinforcing the conclusion that Medicare spending for chronically ill patients in the last two years of life is a reliable proxy for general spending intensity. In addition, there was a moderate positive correlation between both measures of spending and one-year mortality among heart attack patients, again revealing worse outcomes, on average, among patients hospitalized at institutions providing a higher level of care intensity. These results, the authors concluded, revealed something about high-spending hospitals in general:

“This does not mean that all high-cost hospitals are low-quality; some of the higher spending may be devoted to beneficial treatments, but some may well be devoted to unnecessary or cost-inefficient care, such as discretionary use of the hospital as a site of care and more frequent use of physician visits, specialist referrals, diagnostic tests, and minor procedures. And no amount of risk, price, or poverty adjustment—or argument about whether to look forward or back—will correct this problem.” [98]

A study by Cohen et al also examined the relationship between higher hospital spending and survival among acute myocardial infarction patients in Ontario, Canada [99]. While the results appeared to show that higher spending decreased the risk of death for heart attack patients, this effect was attenuated when the researchers accounted for “survivor-treatment selection bias”: the simple fact that, the longer a patient lives—and thus is available to receive treatment—the greater the potential for increased costs. This bias has the potential to overestimate the value of higher spending in terms of survival if not accounted for, and indeed, the study found that, at least for heart attack patients, “the protective effect of spending was overestimated by 62 percent… when the time-varying nature of inpatient cost was not accounted for.” [99] While it is possible that studying care intensity among retrospective cohorts of patients who died might not account for the benefit to those who survived, it is also important to recognize that higher spending might appear to benefit survivors only because they were observed for a longer period.

## Recent studies

More recent research at Dartmouth and elsewhere has continued to use end-of-life measures to explore the influence of both patient factors and practice patterns on unwarranted variation in the treatment of seriously ill patients at risk of dying. In a 2018 prospective observational study, Kelley et al identified patients with a serious medical condition, calculated their one-year mortality risk, and followed them forward for one year. The results showed that, while poorer health status was—as one might expect—associated with high-intensity care in general, for patients estimated to have the highest mortality risk, residence in a region with high end-of-life spending overall was one of the strongest predictors of higher costs for these patients, irrespective of health status; excluding end-stage renal disease, health status was not significantly associated with costs in the highest-risk group. The authors stated: “in the setting of poor prognosis, nonmedical characteristics (i.e., factors likely related to discretionary treatment decisions and not medical need) have greater influences on treatment intensity.” [100]

Cutler et al linked results from patient and physician surveys to Medicare claims in a 2019 study to assess the influence of demand-side (patient) and supply-side (physician) factors in explaining variations in Medicare spending. Physicians were asked to respond to vignette-based questions about how they would manage elderly patients in various scenarios, [89] while a different survey assessed patient preferences [62]. The study concluded that, while patient demand is not a significant predictor of end-of-life spending variations, “35 percent of spending for end-of-life care and 12 percent of spending for heart attack patients (and for all enrollees) is associated with physician beliefs unsupported by clinical evidence.” [89] Both of these studies provide new approaches to measuring care intensity at the end of life and suggest that interventions on the supply side—for example, giving physicians better training to provide care consistent with high-risk patients’ values and goals [100], and better understanding how physicians’ beliefs and practice styles form and could be changed [89]—could be the focus of efforts to improve quality and reduce spending.

More recently, Zhang et al created a new measure of end-of-life care intensity—elevated end-of-life (EoL) spending, defined as the difference in Medicare spending between patients with the same predicted mortality risk who died versus those who survived—to identify potentially wasteful spending on patients that ultimately died [101]. The researchers compared this measure to Dartmouth Atlas end-of-life spending rates at the hospital referral region level. While positively correlated, the two spending estimates had important differences and captured different regional utilization patterns; nevertheless, high expenditures according to the new measure were not found to be associated with better quality, according to selected CMS Hospital Compare measures, or better outcomes for either survivors (receiving expensive but effective treatments) or decedents (receiving the type of end-of-life care they preferred, according to survey data). Again, elevated EoL spending was strongly related to physician practice patterns, particularly the use of the hospital as the site of care, suggesting that “treatment style preferences unrelated to the improvement of quality do drive elevated EoL spending.” [101] Developing prediction models to help physicians more accurately assess their patients’ prognosis and mortality risk in order to reduce wasteful spending on patients who ultimately die, the authors argued, could be another promising area of research.

## The implications of end-of-life studies

Creating risk-adjusted measures of care intensity for seriously ill patients at the regional and hospital levels allowed Dartmouth investigators to focus on two key themes while at the same time reducing the potential for confounding. The first is that capacity and clinician practice styles drive utilization; one of the most important factors in determining whether chronically ill patients are more likely to receive high-intensity hospital care—or to die in a hospital—is the number of available beds per capita. This holds true for patients with different demographic characteristics, illnesses, and levels of disease severity. The second is that more care is not better; patients who receive higher-intensity care do not live longer, experience higher quality care, or express greater satisfaction with their care. Having more medical specialists involved in patient care often results in less effective care management, poor communication, and fragmented, disordered care. End-of-life studies do not show whether a particular intervention is futile, or an individual hospital delivers poor-quality care. Rather, by identifying aggressive, expensive end-of-life care as a measure of exposure to treatment that is often neither needed, wanted, nor effective, Dartmouth researchers created a proxy that could be used to identify regions and hospitals where there were opportunities for improvement.

## Summary

The development of end-of-life cohorts by the Dartmouth Atlas Project provided risk-adjusted measures to show that Medicare patients with chronic illness receiving high-intensity care do not experience higher quality care, greater satisfaction, or better outcomes. The intensity of care delivered to patients at the end of life also closely correlates with overall utilization and spending at the regional level, demonstrating the effects of local factors such as hospital bed capacity, the composition of the physician workforce, and hospital and physician care processes. Regional and hospital-specific variation studies point out places where there are opportunities to improve care and save costs without negative impacts on patients.
